# Commentary on: Trigeminal Neuralgia: Frequency of Occurrence in Different Nerve Branches

**DOI:** 10.5812/kowsar.22287523.3573

**Published:** 2012-01-01

**Authors:** Cyrille Capel, Johann Peltier

**Affiliations:** 1 Department of Neurosurgery, University Hospital of Amiens, Amiens, France

**Keywords:** Trigeminal Neuralgia, Foramen Ovale, Magnetic Resonance Imaging

Dear Editor,

We read this article with great interest regarding the frequency of occurrence in different nerve branches in trigeminal neuralgia (1). However, the paper raises some important issues.

The authors showed that more patients presented with right than left neuralgia (ratio: 1.5/1), attributing this lateralization of neuralgia to a narrower foramen ovale and foramen rotundum on the right side. This hypothesis is to be considered with caution, because some authors have clearly shown robust symmetry between the right and left cranial base foramina size (2). Surprisingly, the vascular conflict hypothesis is not discussed in this paper. Kakizawa et al. noted a 49% rate of vascular contact trigeminal nerves in a magnetic resonance imaging (MRI) study of 220 nerves; the superior cerebellar artery was the most frequent cause of compression (67.5%) (3). In the literature, we have also found descriptions of the venous conflict hypothesis. We know that a long and deeply placed vein of Dandy can be implicated in the etiology of trigeminal neuralgia due to its proximity to the root entry zone, especially if this vein drains predominantly from the pontine area (4). The transverse pontine vein could be a causative factor of venous origin in the pathogenesis of trigeminal neuralgia (5). To complete the vascular conflict hypothesis, regardless of the MRI results (normal or vascular conflict), 40 patients with trigeminal neuralgia underwent microvascular decompression surgery (3), 92.5% of whom were pain-free and did not require any medical treatment. These results demonstrate vascular conflict empiracally and must be seriously explored.

The authors found that the inferior alveolar nerve was involved in 31% of patients. Anil et al. noted a very interesting anatomic variation in 2 of 20 dissections (6). A connecting nerve branch from the auriculotemporal nerve that was joined to the inferior alveolar nerve. The maxillary artery coursed between the root of the inferior alveolar nerve and this connecting nerve branch. This finding can explain the large proportion of inferior alveolar nerve involvement. Your interesting report describes another approach and should be furthered by a parallel vascular study.
